# Diagnosis of latent tuberculosis: Can we do better?

**DOI:** 10.4103/1817-1737.44778

**Published:** 2009

**Authors:** Ibrahim O. Al-Orainey

**Affiliations:** *Department of Medicine, College of Medicine, King Saud University, P. O. Box 2925, Riyadh 11426, Saudi Arabia*

**Keywords:** Tuberculosis, latent, tuberculin test

## Abstract

Latent tuberculosis infection (LTBI) is often diagnosed by the tuberculin skin test (TST). The latter has several limitations with regard to its sensitivity and specificity. It may be positive in people with prior bacille Calmette-Guérin (BCG) vaccination or exposure to nontuberculous mycobacteria. False negative TST results frequently occur in patients with impaired T-cell function. Therefore TST results have to be interpreted taking into consideration the pretest risk of TB infection or reactivation. Recently, interferon gamma release assays (IGRA) were introduced for the diagnosis of LTBI. These include the T-SPOT-TB and the QuantiFERON^®^-TB Gold tests.These tests measure interferon gamma released in response to T-cell stimulation by specific Mycobacterium tuberculosis antigens. These tests have been shown to be more specific than the TST as they are not affected by BCG vaccination. Their sensitivity was similar to that of the TST and in some studies they correlated better with the degree of exposure. In immune-compromised patients their sensitivity was better than that of the TST. IGRA tests were shown to have better predictive value for the development of active disease among individuals with LTBI. These tests are expensive. Their most cost-effective utilization is as confirmatory tests in patients with positive TST results, particularly in areas with high rates of BCG vaccination.

There are two billion people infected with *Mycobacterium tuberculosis* all over the world.[1] The majority of these infections are asymptomatic (latent) and are detected by a positive tuberculin skin test (TST). These latent infections may reactivate later in life. This large pool of latent tuberculosis (TB) constitutes an important source of infection.

Longitudinal studies have shown that among people with positive tuberculin test and no other risk factors, the likelihood of developing active TB is about 0.1% every year.[2] The risk is higher in certain situations. Close contacts of infectious TB patients who become tuberculin positive (i.e., converters) have a 5–10% risk of developing active TB in the following 2–5 years and another 5–10% during their lifetime.[3] Other factors that increase the risk of reactivation of latent TB include HIV infection; old TB, with lung scarring; immunosuppression; organ transplantation; malignant disease; end-stage renal failure; and diabetes mellitus.[4]

Diagnosing latent tuberculosis infection (LTBI) is important for the overall control of the disease. Offering antituberculous treatment to individuals with LTBI significantly decreases their risk of developing active tuberculosis.[5] For years, the diagnosis of LTBI infection relied on the TST. The latter is known to have several limitations compromising its sensitivity and specificity. Recently immune-based blood tests were developed with the hope of improving the diagnosis of LTBI. This paper will review the performance of these new tests: their sensitivity and specificity and their role in the diagnosis of latent TB.

## Diagnosis of Latent Tuberculosis

LTBI is a subclinical infection with *M. tuberculosis* without clinical, bacteriological, or radiological evidence of the disease. The standard test for diagnosis of LTBI is the TST. This involves the intradermal injection of purified protein derivative (PPD), which leads to a delayed-type hypersensitivity response causing a cutaneous induration at the site of injection which peaks at 48-72 h. PPD is a mixture of more than 200 antigens that are also shared by other mycobacteria. A positive TST indicates previous exposure to *M. tuberculosis* or other nontuberculous mycobacteria or prior BCG vaccination.[6–9] The latter is routinely included in the immunization regimen and is given at birth or in infancy in many countries, including in Saudi Arabia. Twelve weeks after bacille Calmette-Guérin (BCG) vaccination, 90% of individuals develop tuberculin induration of ≥ 10 mm.[10] The reaction usually wanes within 1 year if the vaccination was given in infancy.[11] However, if BCG was given after the first year of life, tuberculin reactions may persist for 1–5 years in the majority of recipients.[12] In some instances, tuberculin reactivity may last for up to 15 years after vaccination.[7] Several studies have shown that BCG-vaccinated individuals of any age are more likely to have positive TST results.[9,13] The booster phenomenon is more common after BCG vaccination, with the tuberculin test becoming positive in 20% of individuals if the test is repeated within 1–4 weeks.[14] Repeated testing (e.g., in health care workers) leads to stronger tuberculin reactions.[15] The low specificity of TST, especially in BCG-vaccinated people, puts into question its use as a “standard” test to diagnose latent TB. The absence of a gold standard to diagnose LTBI makes it difficult to estimate the exact sensitivity and specificity of TST. TST may also be falsely negative in patients with impaired T-cell function (e.g., in those with HIV infection or immunosuppression). Such patients are at high risk of developing TB, but the poor sensitivity of TST limits its utilization in these situations.[16]

Tuberculin skin test interpretation may be affected by the PPD dose and by operator variability in both inoculation and reading.[17] A low PPD dose (e.g., 2 U) may lead to a false negative reaction and a high dose (e.g., 10 U) may cause a false positive result.[18] There is no international consensus on what constitutes TST positivity. Different cutoffs are used in different countries. In the USA, an induration of ≥ 5 mm is considered positive in contacts of infectious TB patients, whereas in the UK an induration of ≥ 15 mm is considered positive for BCG-vaccinated contacts.[4,19]

To overcome the limitations of the TST, the American guidelines for interpretation of TST were revised to include the pretest risk of TB infection or reactivation.[4] An induration of ≥ 5 mm is considered positive in patients with a high risk of infection or reactivation (e.g., recent contacts of infectious cases or HIV infection). An induration of ≥ 10 mm is considered positive for those with intermediate risk (e.g., residents of long-term facilities or patients with chronic diseases). For those with no risks, an induration of ≥ 15 mm is considered positive. These guidelines ignored the effect of BCG vaccination when interpreting the TST.[4]

Despite its shortcomings, TST remains in widespread use simply because of the lack of better alternatives. This highlights the need to continue the search to find more accurate diagnostic tests for latent TB.

## New Alternative Tests

In the absence of a gold standard to diagnose LTBI, it may be difficult to demonstrate that any test is better than the TST. However, the sensitivity of a potential test may be predicted by correlating its results with the degree of exposure (duration and proximity) to a source patient and the likelihood of acquiring infection from that source. A test would be more sensitive than the TST if it is positive in patients with a high risk of exposure. A more specific test would be independent of BCG vaccination.

Recently new immune-based blood tests were developed for the diagnosis of TB. These measure interferon gamma released in response to stimulation of sensitized T-cells by mycobacterial antigens.[20] The first commercially available test was QuantiFERON^®^ TB assay; which measures interferon gamma production by ELISA after *in vitro* stimulation of white blood cells with PPD.[21] It was found to have sensitivity comparable to that of TST. It may give false positive results in BCG-vaccinated people and in those exposed to nontuberculous mycobacteria.[22]

Comparative genomic studies of mycobacteria identified a genomic region in *M. tuberculosis* that is not present in BCG strains and in most nontuberculous mycobacteria.[23] This so-called region of difference 1 (RD1) encodes antigens that are highly specific for *M. tuberculosis*. The best studied of these are ESAT-6 and CFP10.[24] These antigens were used to develop more specific T-cell–based tests for the diagnosis of TB infection. There are two commercially available diagnostic tests incorporating specific antigens. QuantiFERON^®^-TB gold test (Cellestis Ltd., Australia) and T-SPOT-TB assay (Oxford Immunotec, UK).[25,26]

The specificity of interferon gamma release assays (IGRA) was studied in healthy low-risk individuals with BCG vaccination. QuantiFERON^®^-TB test was evaluated in 216 Japanese nursing students with no risk factors for TB exposure. All of them had had BCG vaccination. TST was positive (≥ 10 mm) in 64.6% (specificity 35.4) while QuantiFERON^®^-TB test was positive in 1.9% (specificity 98.1%).[27] A Korean study evaluated QuantiFERON^®^-TB Gold in 99 healthy adults with no risks for TB.[28] The majority (90%) of them were BCG vaccinated. QuantiFERON^®^-TB was positive in only 4% (specificity 96%) while TST was positive in 51% (specificity 49%). Another study found that QuantiFERON^®^-TB was negative in all 50 healthy medical students (74% BCG vaccinated).[29] TST was positive in 36% of them. These studies and others demonstrate clearly that IGRA tests are significantly more specific for the diagnosis of LTBI than the TST [Table 1].[30–35]

**Table 1:** Studies on specificity of interferon gamma assays in healthy BCG-vaccinated individuals

	No.	TST +ve	IGRA +ve	Specificity of IGRA (%)
Lalvani[30]*	36	ND	1	97
Pathan[32]*	28	ND	0	100
Chapman[31]*	33	ND	0	100
Brock[33]**	22	ND	0	100
Mori[27]**	216	113	4	98
Ravn[34]**	39	ND	0	100
Kang[28]**	99	51	4	96
Kobashi[29]**	37	18	0	100

ND – Not done,

* T SPOT- TB Assay,

** Quantiferon - TB Gold assay

The sensitivity of IGRA tests was studied in patients with active TB and contacts of infectious TB patients. In patients with active TB, IGRA tests were found to have a sensitivity of 74–96%, while TST had a sensitivity of 64–69% in this population.[28–30]

In contact-tracing studies, IGRA tests were found to be as sensitive as TST for LTBI and, in some studies, they correlated better with the degree of exposure.[36–38] They also showed high specificity in BCG-vaccinated contacts. In the largest of these studies, the T-SPOT-TB test was evaluated in 535 secondary school students who had been exposed to an infectious TB case.[36] Most of them were BCG vaccinated. T-SPOT-TB test correlated more strongly than TST with the degree of exposure (closeness and duration of contact), indicating the superior sensitivity of the test. It also was not affected by BCG vaccination status. QuantiFERON^®^-TB Gold was evaluated in contact tracing of 85 BCG-unvaccinated individuals.[39] Its sensitivity was found to be similar to that of TST. These findings were confirmed by other investigators [Table 2].[40–43]

**Table 2:** Studies on sensitivity of interferon gamma assays in contacts of infectious TB patients

	Close contacts (high exposure)			Casual contacts (low exposure)			BCG (all) %
			
	No.	TST + (%)	IGRA + (%)	No.	TST + (%)	IGRA + (%)	
Lalvani[37]*	22	65	72	20	32	0	82
Ewer[36]*	101	58	62	386	20	17	87
Brock[39]**	45	53	55	40	10	5	0
Kang[28]**	48	71	44	72	60	10	81
Shams[40]*	104	57	50	103	41	30	49
Zellweger[41]*	54	50	22	37	35	13	86
Hill[42]*	163	32	28	182	8	10	46
Nakaoka[38]**	72	53	74	39	15	10	90
Diel[43]**	124	23	17	232	9	1	46

* T SPOT - TB assay,

** QuantiFERON^®^- TB Gold assay, TST - Tuberculin skin test, IGRA - Interferon gamma release assays, BCG - Bacille Calmette-Guérin

In immunocompromised patients, the data is too scarce to make any definite conclusions. In a study of 39 HIV-positive patients with TB, the sensitivity of the T-SPOT-TB test was found to be 92%.[31] TST was not done in this group but it is known that its sensitivity is low in this population. Another study of 590 HIV-infected patients showed that QuantiFERON^®^ -TB Gold test correlated with known risk factors for LTBI or past history of TB.[44] HIV-positive patients with low CD4 counts were more likely to have indeterminate QuantiFERON^®^ results.

A few studies have looked at the predictive value of IGRA tests for future development of TB.[45] The gold standard proof of LTBI is the eventual development of active disease. This can only be evaluated by longitudinal cohort studies that follow tested individuals for development of TB. Diel *et al.*, in Germany, evaluated 601 close contacts of infective TB patients of whom 278 (46.3%) had had BCG vaccination.[43] TST was positive in 243 (40%), while QuantiFERON^®^ -TB Gold was positive in 66 (11%) contacts. Isoniazid was offered only to contacts with positive QuantiFERON^®^-TB test. Forty-one contacts declined to take isoniazid. All contacts were followed for 2 years. Six of contacts developed active TB during follow-up and all six were QuantiFERON^®^ positive. None of the QuantiFERON ®-negative individuals developed TB. This important study indicates that QuantiFERON^®^-TB Gold is a more accurate indicator for LTBI and a better predictor for the development of TB. In another study, 88 TST-positive contacts of an index case were followed for 3.5 years. Only four of them had positive QuantiFERON^®^-TB test. None of the 84 QuantiFERON^®^-negative contacts developed TB during follow-up. This clearly confirms the high specificity and predictive value of the QuantiFERON^®^-TB Gold assay.

IGRA tests have several operational advantages over TST. They require only one visit for blood sampling. Automated reading reduces the reader bias in interpretation. There is no booster effect of the test and, therefore, repeated testing (e.g., in health care workers) does not affect results. The test is read within 24 h. IGRA tests require at least a basic laboratory and some technical skills. Blood need to be processed within 6 h of venipuncture. Storing samples for longer periods makes results less reliable. The development of QuantiFERON^®^ in-tube assay is likely to overcome this problem.

## Role of IGRA Tests in the Diagnosis of LTBI

The main advantage of IGRA tests is their high specificity compared to TST. This significantly eliminates false positive results in BCG-vaccinated individuals and therefore avoids the costs and toxicity associated with unnecessary treatment. The sensitivity of IGRA tests is similar to that of TST. In contact-tracing studies, they showed good correlation with the degree of exposure to an index case. In some studies, sensitivity of the IGRA tests was better, particularly among immune-compromised patients. Detection of LTBI in these patients is highly important because of their increased risk of progression to active disease.

As mentioned previously, IGRA tests have several operational advantages. Their main disadvantage, however, is their high cost. A single assay usually costs around $30–40. The cost to the healthcare system may initially increase but the overall cost will decrease, as less LTBI patients are treated and less visits are required. The costs are likely to decline with increasing usage of these tests. Both T-SPOT-TB and QuantiFERON^®^-TB Gold tests are approved in Western Europe for the diagnosis of LTBI. QuantiFERON^®^-TB Gold is also approved by the USA Food and Drug Administration (FDA).[46]

The question is whether IGRA tests should complement or replace TST. The latter test is cheap and sensitive for diagnosis of LTBI. However, in BCG-vaccinated individuals, its specificity is clearly inferior to that of the IGRA tests. These expensive tests may not be affordable to many developing countries. Therefore, it is likely that TST will remain in use in many parts of the world.

In resource-rich countries, IGRA tests are increasingly utilized. In their guidelines, the Center for Disease Control and Prevention (USA) have suggested replacing TST by QuantiFERON^®^-TB Gold.[46] The objective is to have only one system in place and to improve the sensitivity and specificity of testing for LTBI. On the other hand, British guidelines recommend using IGRA tests as confirmatory tests in those with positive TST results.[19] In their evaluation, they found that this two-step approach is the most cost-effective. They also recommended using IGRA tests in situations where TST may not be reliable (e.g., in immune-compromised patients). A cost–analysis study in Germany found that screening of contacts by TST followed by QuantiFERON^®^-TB Gold assay in positive TST reactors was the most cost-effective method for screening for latent TB.[47]

In countries where the BCG vaccination rate is high (as in Saudi Arabia), the two-step approach is probably more efficient for screening of high-risk individuals (e.g., contacts of infectious patients, health care workers, etc). It improves the diagnostic accuracy and reduces false positive results. This is likely to have a significant positive impact on the control of latent TB.

Interferon gamma assays are an important step in the search for better diagnostic tests for latent TB. However, more research is required to improve their performance, especially in immunocompromised patients. Longitudinal studies are also needed to assess the value of these tests in predicting the development of active TB and to demonstrate clearly their superiority to the TST.
